# Investigating Meta-Approaches for Reconstructing Gene Networks in a Mammalian Cellular Context

**DOI:** 10.1371/journal.pone.0028713

**Published:** 2012-01-09

**Authors:** Azree Nazri, Pietro Lio

**Affiliations:** 1 Department of Computer Science, Faculty of Computer Science & Information Technology, University Putra Malaysia, Malaysia, Selangor, Malaysia; 2 Department of Applied Mathematics and Theoretical Physics, University of Cambridge, Cambridge, United Kingdom; 3 Computer Laboratory, University of Cambridge, Cambridge, United Kingdom; 4 Department of Physiology, Development and Neuroscience, University of Cambridge, Cambridge, United Kingdom; I2MC INSERM UMR U1048, France

## Abstract

The output of state-of-the-art reverse-engineering methods for biological networks is often based on the fitting of a mathematical model to the data. Typically, different datasets do not give single consistent network predictions but rather an ensemble of inconsistent networks inferred under the same reverse-engineering method that are only consistent with the specific experimentally measured data. Here, we focus on an alternative approach for combining the information contained within such an ensemble of inconsistent gene networks called meta-analysis, to make more accurate predictions and to estimate the reliability of these predictions. We review two existing meta-analysis approaches; the Fisher transformation combined coefficient test (FTCCT) and Fisher's inverse combined probability test (FICPT); and compare their performance with five well-known methods, ARACNe, Context Likelihood or Relatedness network (CLR), Maximum Relevance Minimum Redundancy (MRNET), Relevance Network (RN) and Bayesian Network (BN). We conducted in-depth numerical ensemble simulations and demonstrated for biological expression data that the meta-analysis approaches consistently outperformed the best gene regulatory network inference (GRNI) methods in the literature. Furthermore, the meta-analysis approaches have a low computational complexity. We conclude that the meta-analysis approaches are a powerful tool for integrating different datasets to give more accurate and reliable predictions for biological networks.

## Introduction

Gene expression microarrays yield quantitative data about the intricate biological processes in cells. They give a systematic understanding of the cell status under specific conditions and at a specific time when inferred by gene regulatory network inference (GRNI) methods. The approaches for inferring gene networks can be classified into two broad categories [1]: those based on physical interactions and those based on influence interactions. The former category deals with identifying interactions among transcription factors and their target genes (gene-to-sequence interactions) whereas the latter category attempts to relate the expression of a gene to the expression of other genes (gene-to-gene interactions). In this study, we refer to GRNI methods as ‘influence interactions’ approaches.

Other expression measurements can also be utilised for experimental detection of biological interaction networks. The most common of these methods, two-hybrid system, uses a physical interaction approach. However, the two-hybrid system has been criticised for having a high false-positive discovery rate [2]. Mass spectrometry has been successfully adapted for large-scale identification of gene and protein complexes [3]. Unfortunately, low correspondence among the different high-throughput interaction studies requires further investigation both computationally and experimentally. Furthermore, a comparison of two-hybrid and mass spectrometry experiments in yeast discovered a relatively small overlap of 387 interactions between the two approaches [4], and similar comparisons by [2] found relatively little correspondence between the studies. These results highlight the need for a computational approach that integrates the inconsistent information from variable high-throughput studies.

In microarrays alone, multiple interaction experiments in budding yeast, for the most part, have revealed different interactions. In such a case, a series of gene expression microarray datasets on the same phenomenon, such as in the same cell under the same condition and at the same time, often contain different levels of noise from both technical and biological factors. If analyzed by a single GRNI method, those datasets might give many inconsistent networks that are only consistent with the specific experimentally measured data. One reason for this inconsistency is that many GRNI methods are based on the fitting of a mathematical model to a specific dataset. Therefore, the outputs produced by a single GRNI method from different gene expression microarray datasets are often not single consistent network predictions but an ensemble of inconsistent networks. One method to alleviate this inconsistency and to form more accurate and reliable predictions is to integrate the inconsistent networks or to identify a unique ‘best’ network from this inconsistent ensemble according to additional criteria [5]–[7]. Moreover, the problem of how to optimally analyse the ensemble of inconsistent gene networks from multiple datasets to estimate the true structure of the underlying gene network has received relatively little attention, although for differentially expressed gene applications, many methods have been proposed to utilise such ensemble techniques [8]–[11].

Recently, many single GNRI methods have been proposed for biological networks; each offers its own unique disadvantages and advantages. Methods such as Boolean networks [12] and Bayesian networks [13] use a mathematical model for inferring gene networks. Other methods such as relevance networks [14], genetic algorithms [15], and neural networks [16] utilise static or dynamic, continuous or discrete, linear or non-linear, and deterministic or stochastic approaches. The latest approaches such as Self-Adaptive Differential Evolution [17], Stepwise Network Inference (SWNI) [18] and parallel island evolutionary strategy [19] adapt learning models based on a linear time-variant approach. Nonetheless, the best of our knowledge, none of these approaches can integrate inconsistent networks from multiple sources. Instead, the GNRI methods produce inconsistent networks when applied to different microarray datasets. Here, we focus on an approach for integrating biological networks called meta-analysis, which aims to integrate the information contained within ensembles of inconsistent networks inferred by a single GNRI from multiple gene expression microarray datasets to give more accurate and reliable predictions for biological networks.

The paper is organised as follows. In the next section, we first justify the use of meta-analysis approaches for biological networks and then formalise the approaches. We describe our simulation set-up and the expression data we used for biological datasets. We present numerical results analysing the performance of the FTCCT, FICPT, ARACNe, CLR, MRNET, relevance network (RN) and Bayesian network (BN) methods in terms of area under the curve (AUC) and tIDR (true integration discovery rate) as well as tIRR (true integration reversion rate) as an accuracy benchmark. We use reproducibility measured by correspondence at the top (CAT) plots, as a reliability indicator. We finish this article with the Discussions.

## Methods

How can we process an ensemble of inconsistent gene networks to obtain an estimate of the true gene network? Let us assume that we have a gene regulatory network of *N* genes called the target gene network. This target gene network is typically represented by an *NxN* weight matrix *B*. The entries *b_ij_* of this matrix give the strength of the regulatory effect of gene *j* on gene *i*. We possess a series of *Z* microarray datasets concerning the activity of the network, from which a reverse-engineering method infers an ensemble of tentative networks. Each network has an associated score *s_k_* that indicates how well it fits the data. Thus, the ensemble is a collection *C = {(B_k_,s_k_)}* where *B_k_* is a plausible network *k*th in ensemble with the highest score *s_k_*. To restate the problem, we consider how to process the ensemble *C* to obtain an estimate of the ‘true’ weight matrix *B*. In this paper, we show that in practice even simple meta-analysis approaches often allow an improvement the accuracy of an ensemble *C* compared to individual inferred networks of the ensemble.

### Combined statistical approaches

The general meta-analytic framework assumes that *N* independent studies have been conducted; in the case of biological networks, these studies would have focused on the relationships between genes. Furthermore, it assumes that a measurable relationship exists between certain quantities of interest such that the relationship can be quantified and each study produces an estimate of the relationship. This estimation is termed an ‘effect size’ if the estimates are appropriately standardised. Thus, an effect size is essentially a standardised quantitative expression of the relationship of interest. In general, there are three main classes of effect size estimates. The first class is the standardised difference estimate, such as Hedge's *g*. The second class is the standardised relation estimate such as the sample correlation coefficient *r*, and the last class is the measure of significance, such as the p-value from a particular hypothesis test.

To combine results across samples or datasets, effect size estimates must address the same measure or quantity, be standardised, and include some measurement of the variability of the effect size estimate. After each study has provided its effect size estimate and a measurement of its variability, a meta-analysis can be performed. In this paper, we have chosen two representations from the second and the third classes of effect size estimates: Fisher's inverse combined probability test (FICPT), which uses a test of significance as its effect size [20] and the Fisher transformation combined correlation test (FTCCT), which uses the correlation coefficient as its effect size [21]. The simplest meta-analysis approach is FICPT, which combines the p-values from independent datasets. One approach for combining the p-values [20] is the Fisher summary statistic,
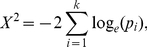
(1)which tests the null hypothesis that, there is no correlation between given pair of genes. The *p_i_* is the p-value for the correlation between *i*th and *j*th genes from the *k*th dataset. The theoretical null distribution should be a chi-squared distribution, *X^2^*. On the other hand, instead of using a test of significance for combining the results, FTCCT uses correlation coefficients as its effect size. The individual correlation estimates are first converted to z-values using Fisher's r-to-z transformation.
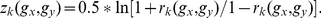
(2)This statistic is approximately normally distributed with variance 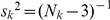, where *N_k_* is the profile length in experiment *k* [22]. The similarity is estimated using the weighted average
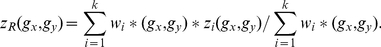
(3)The weights are defined as 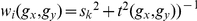, where the coefficient 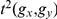 is estimated using Cochran's Q-statistic as described by [23]. Finally, Fisher's z-to-r transformation is applied to convert the results back into correlations.
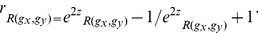
(4)After the transformation it is reasonable to check the significance of the effect size estimate. Even if the variables have no correlation, for samples of finite size, the correlation coefficient will be non-zero. There are two ways to test the significance for FTCCT. The first method uses a test statistic t:
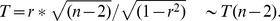
(5)This test statistic is distributed approximately according to a t-distribution. The correlation coefficient is considered to be statistically significant if the computed t value is greater than the critical value of a t-distribution with a level of significance of α. The second test of significance is a z-test that assumes the gene profile (*X,Y*) has a jointly bivariate normal distribution. Thus, the correlation coefficient can be transformed such that the transformed statistics conform to the normal distribution as follows
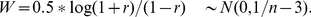
(6)


### Reverse-engineering methods

Thus far several methods have been suggested for inferring gene regulatory networks [24]–[26]. For comparison, we chose ARACNe, CLR, MRNET, Bayesian network (BN), and relevance network (RN). All the parameter settings are discussed in the Supplementary Information S2, and the software is freely downloadable. For detailed information about these inferring algorithms considered in this study, we refer the reader to [27], [28], [29], [30], and [31]. In this subsection, we discuss the correlation coefficient used in both FICPT and FTCCT. Gene expression is recorded as an *nxn* matrix with *n* genes, each of which has *m* experimental conditions or time points. We used the partial correlation coefficient *r* as the pair-wise measure of the linear relationship between two gene profiles. A partial correlation coefficient quantifies the correlation between two variables when conditioning on one or several other variables. Partial correlation can be calculated as follows
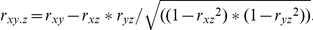
For FICPT, the partial correlation coefficients *r* are first converted to p-values before they are combined using (1). For FTCCT, the partial correlation coefficients *r* are first combined before they are converted to p-values.

### Simulated and experimental datasets

For analysing and comparing meta-analysis approaches (FTCCT and FICPT) together with single inferring methods (ARACNe, CLR, MRNET, BN and RN), we utilised simulated and experimental expression datasets from microarray experiments. Simulated microarray data are needed because the knowledge about biological regulatory networks is still incomplete and imperfect. Simulated data are used because, for these data, we know the true regulatory network precisely. This knowledge allows a detailed and accurate analysis. The experimental microarray datasets are used to demonstrate the reliability of meta-analysis approaches over single inferring methods and to show that the assumptions made for simulated datasets are sufficiently valid.

**Figure 1:**
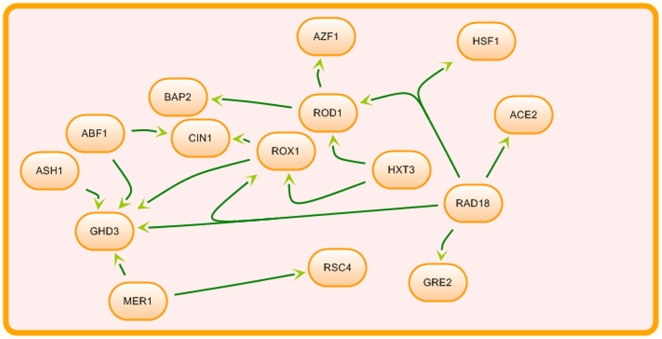
Sub-network generated by SynTReN based on scale-free topology using a Saccharomyces cerevisiae source network containing 15 genes and 17 edges.

All simulated data are generated based on a scale-free network. It has been shown that the network presents a difficult challenge for reconstruction algorithms. The challenges present in the network are important to approximate the true network in experimental cells, which have a few highly interconnected genes, and the biologically motivated non-linear transcriptional dependencies among genes [32]. It is highly unlikely that any inferring method that does not perform well on this network could withstand a more complex case. To generate a synthetic network, we utilised Synthetic Transcriptional Regulatory Networks (SynTRen) by [33]. The gene network is shown in Figure 1. All simulations and settings are described in the Supplementary Information S1.

For experimental datasets, we used two different cancer samples: breast cancer and colorectal cancer. The colorectal cancer datasets consist of three datasets obtained from a public repository (NCBI) with 62 samples for the first dataset (accession code GSE 12945), 55 samples for the second dataset (accession code GSE17357) and 73 samples for the third dataset (accession code GSE 13067). The breast cancer expression datasets consist of three datasets with 266 samples for the first dataset (accession code GSE 21653), 51 samples for the second dataset (accession code GSE 17907) and 55 samples for the third dataset (accession code GSE 16391).

### Benchmarking tools

It is important to assess the performance and reliability of meta-analysis approaches and single reverse-engineering methods. In this paper, we evaluated the performance and reliability of the single reverse-engineering methods and the meta-analysis approaches. For the performance evaluation, we use area under curve (AUC), true integration discovery rate (tIDR) [23] and true integration revision rate (tIRR) [34].

We defined tIDR as the true associations identified in the meta-analysis (FTCCT and FICPT) that were not identified in any of the separate studies alone (ARACNe, CLR, MRNET and RN). We fixed the statistical threshold to filter any insignificant associations, α = 1e-12, and labelled genes as correlated if (p_i_<α), p_i_ as p-value.
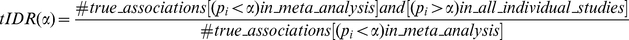



On the other hand, tIRR can be defined as the proportion of true pairs of genes that were declared correlated in at least one separate study but not in the meta-analysis,
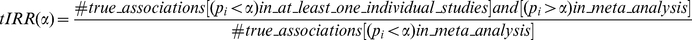



In addition to the aforementioned performance indicators, we also utilised ensemble evaluations that make statistical statements about the individual edges or motifs of underlying simulated networks structure inferred by either single reverse-engineering methods or meta-analysis approaches to reveal and investigate their strengths and weaknesses. These ensemble evaluations have been shown to be good explanatory tools for investigating inference algorithms on the level of network components, such as edges, motifs or sub-networks [35]. The statistical statements of characteristics of a network structure of an inferring method could give an insight into why AUC meta-analysis approaches are better than single reverse-engineering methods. More details can be found in [35].

The reliability of the single reverse-engineering methods and meta-analysis approaches was also evaluated for experimental datasets using reproducibility measured by CAT plot [36]. It is important to assess an agreement for interactions that are likely to be called significant because interactions identified in multiple independent studies are likely to be the truly significant ones. Thus, high reproducibility among independent studies suggests a high specificity. CAT plots can be used as a reliability assessment tool for assessing the agreement of the identification among studies. CAT plots were originally developed for differentially expressed gene applications, where a list of *n* top candidate differentially expressed genes for each of two studies is used. For biological network application, we modified the list of genes from a hub to accommodate the different outcomes for gene regulatory networks. CAT plots were produced by the same procedure as in differentially expressed gene applications [36].

## Results

Because reverse-engineering methods (ARACNe, CLR, MRNET, BN and RN) and meta-analysis approaches (FICPT and FTCCT) are used in different contexts, we divided performance evaluation into three benchmarks but used only one benchmark for reliability evaluation. The first performance benchmark is for analysing the AUCs of meta-analysis approaches and the single reverse-engineering methods; the second benchmark is to measure the power of single reverse-engineering methods and meta-analysis approaches using tIRR and tIDR; and the last benchmark is for ensemble evaluations.

### Performance evaluations

#### Benchmark for performance evaluation #1: Which meta-analysis approaches *have significant* differences in performance compared to single reverse-engineering methods

**Figure 2:**
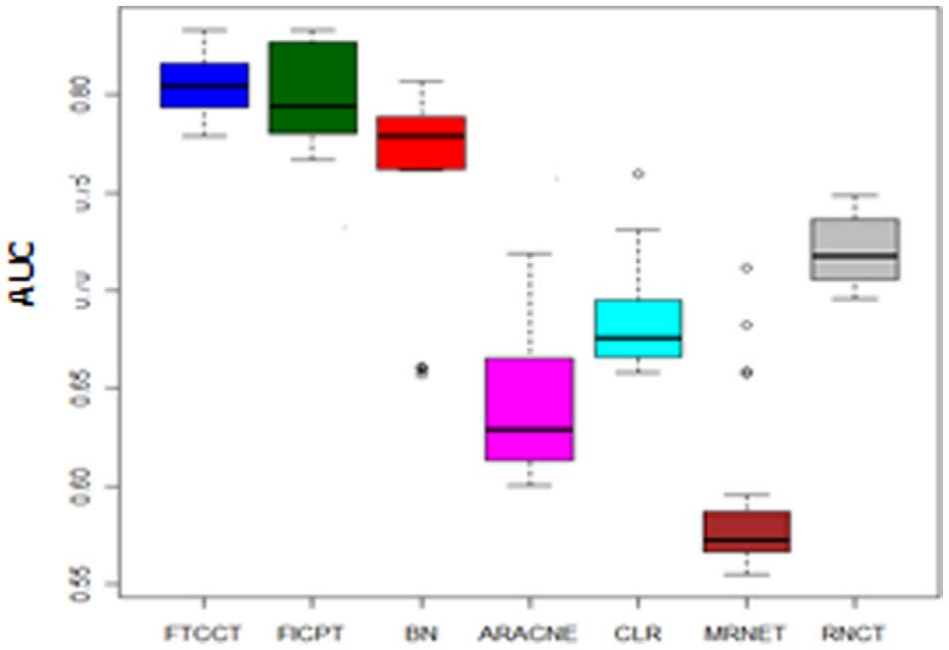
AUCs of FTCCT, FICPT, BN, ARACNe, CLR, MRNET and RN. RNCT represents RN. Boxplots for FTCCT (blue), FICPT (dark green), BN (red), ARACNE (magenta), CLR (cyan), MRNET (brown) and RN (grey). A subnetwork of yeast is used for the simulations. The sample size is 1000 and the ensemble size is 24.

We first analysed the performance of the single reverse-engineering methods and meta-analysis approaches in terms of their sensitivity-specificity measurements using AUC. Fig. 2 shows the boxplots of the resulting AUCs for FTCCT, FICPT, BN, ARACNe, CLR, MRNET and RN for an ensemble size of *N* = 24. The yeast gene network generated by SynTReN used as the underlying network structure is shown in Fig. 1. From Fig. 2, overall, one can see that the meta-analysis approaches provided better results than ARACNe, CLR, MRNET, RN and BN, as indicated by the median values of the AUC of each boxplot. The boxplots show that, statistically, FTCCT with a median AUC of ∼0.80 is better than BN with a median AUC score of ∼0.77, ARACNe with a score of ∼0.63, CLR with a score of ∼0.67, MRNET with a score of ∼0.56 and RN with a score of ∼0.73. FICPT with a median AUC of ∼0.79 also shows a better AUC median score than ARACNe, CLR, MRNET and RN, and the score is comparable to that of BN. A summary of the AUCs for FTCCT, FICPT, ARACNe, CLR, MRNET, RN and BN is provided in Table 1 and details in Supporting Information S3. Table 1 suggests that, for a significance level of α = 1e-05, FTCCT has a statistically significant performance result compared to BN (p-value = 9.424e-07), RN (p-value = 6.609e-09), ARACNe (p-value = 6.609e-09), MRNET (p-value = 3.591e-08) and CLR (p-value = 3.591e-08). FICPT has a statistically significant performance result at a significance level of α = 1e-03 compared to BN (p-value = 2.097e-03), RN (p-value = 6.609e-09), ARACNe (p-value = 6.609e-09), MRNET (p-value = 6.609e-09) and CLR (p-value = 6.609e-09).

**Table 1:** Test of significance of the AUC metric for single reverse-engineering methods and meta-analysis approaches from 24 simulated datasets.

AUC					
Methods	BN	RN	ARACNe	MRNET	CLR
FTCCT	*9.424e-07*	*6.609e-09*	*6.609e-09*	*3.591e-08*	*3.591e-08*
FICPT	*2.097e-03*	*6.609e-09*	*6.602e-09*	*6.602e-09*	*6.602e-09*

p-values in bold-italic indicate meta-analysis approaches with significantly better AUCs than the single reverse-engineering methods.

#### Benchmark for performance evaluation #2: Which meta-analysis methods have significantly different discovery rates compared to the single reverse engineering methods

The parameter tIDR measures the power of a meta-analysis approach over a single reverse-engineering method, while tIRR measures the associations missed by the meta-analysis. In other words, tIDR measures the power of meta-analysis approaches, and tIRR measures the power of the single reverse-engineering methods. We used 1e-12 as the statistical threshold for producing tIDR and tIRR. All twenty-four datasets were combined to create cumulative samples using either concatenation or meta-analysis formulae. The concatenated method is used to combine multiple datasets into a single dataset with copula-transform. The resulting single dataset is then submitted to the reverse-engineering methods for inferring. Copula-transform is a technique for concatenating individual datasets into a single dataset that is widely used in the reconstruction of gene networks (Basso et al., 2005). The inferred results from this single dataset are then used for analysis. FICPT and FTCCT gave significantly better results than ARACNe, CLR, MRNET, RN and BN, as shown in Table 2. From Table 2 and details in Supporting Information S4, for a significance level of α = 1e-05, FTCCT is better than RN (p-value = 3.122e-05), ARACNe (p-value = 2.817e-09), MRNET (p-value = 2.887e-09), and CLR (p-value = 1.119e-08). At the same significance level, FICPT shows improvement over RN (p-value = 4.921e-09), ARACNe (p-value = 1.775e-09), MRNET (p-value = 1.775e-09) and CLR (p-value = 8.282e-08). We dropped BNs because to make a reliable comparison, the output from this method needs to be converted from a probability value to a p-value and it was safer to forgo analysis to avoid biased results.

**Table 2:** tIDR-tIRR metric for RN, ARACNe, MRNET and CLR, FTCCT and FICPT from 24 simulated datasets.

tIDR-tIRR				
Methods	RN	ARACNe	MRNET	CLR
FTCCT	*3.122e-05*	*2.817e-09*	*2.887e-09*	*1.119e-08*
FICPT	*4.921e-09*	*1.775e-09*	*1.775e-09*	*8.284e-08*

p-values in bold-italic indicate cases in which tIDR is significantly better than tIRR.

#### Benchmark for performance evaluations #3: Are there any statistical similarities or differences between MAs and RNCT

**Figure 3:**
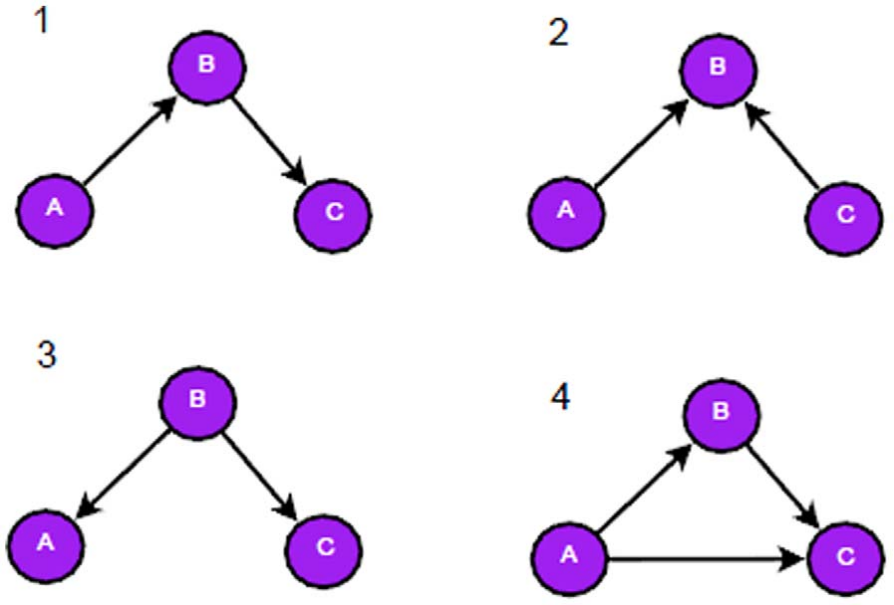
Directed network motifs consisting of three genes. 1) chain, 2) collider, 3) fork, and 4) triangle.

In this section, we study two ensemble evaluations. The first ensemble evaluation measures the ability to infer basic motif types that consist of four different three-gene motifs. The motifs are shown in Fig. 3 and discussed in detail in [35]. The second ensemble evaluation analyse the behaviour of individual edges. Here, we only compare FTCCT and FICPT to RN, as these methods use the same effect size, which is equal to the correlation coefficients.

Table 3, details in Supporting Information S5, shows the results for the first ensemble evaluation using four network motifs for a sample size of 24, providing their mean true reconstruction rate *p*. Briefly, to calculate *p*, for motifs of types 1, 2 and 3 for example, is given by averaging
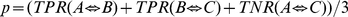
over all motifs of the same type within the network (Emmert-Streib and Altay, 2010). In this equation, TPR represents the true positive rate, and TNR represents the true negative rate. Table 3 suggests that FICPT and FTCCT behave similarly in favouring all motif types with a mean true reconstruction rate greater than 0.5. The mean true reconstruction rate for RN is the lowest (below 0.5) for types 1 and 2. However, as one can see from Table 4, all mean true reconstruction rates for motif type 3 are larger than 0.5 for FTCCT, FICPT and RN. The results suggest that no bias is introduced by FTCCT and FICPT regarding the ability to infer individual motif types. However, RN is biased toward motif type 3. There is no motif type 4 in the simulated gene network.

**Table 3:** Summary of motif statistics for FTCCT, FICPT and RN.

Measure/motif type	1	2	3	4
*#m*	*5*	*10*	*18*	*0*
***FTCCT (p)***	0.6927536	0.668116	0.5716586	0
***FICPT (p)***	0.7275362	0.5318841	0.5764896	0
***RN (p)***	0.3942029	0.2536232	0.510467	0

**Table 4:** Summary statistics for leaf and hub edges for FTCCT, FICPT and RN.

FTCCT				
*Measure/edge type*	*Red*	*Green*	*Blue*	*Black*
True network (#)	6	1	0	10
True network (%)	35.3	5.89	0	58.8
Leaves (#)	1	0	0	6
Leaves (% E_L_)	14.3	0	0	85.7
Leaves (% N_et_)	5.88	0	0	35.3
In-hubs (#)	1	0	0	6
In-hubs(% E_L_)	12.5	0	0	75
In-hubs(% N_et_)	5.88	0	0	35.3

FICPT				
*Measure/edge type*	*Red*	*Green*	*Blue*	*Black*
True network (#)	5	1	1	10
True network (%)	29.4	5.88	5.88	58.8
Leaves (#)	1	0	0	6
Leaves (% E_L_)	14.3	0	0	85.7
Leaves (% N_et_)	5.88	0	0	35.3
In-hubs (#)	1	0	0	4
In-hubs(% E_L_)	12.5	0	0	50
In-hubs(% N_et_)	5.88	0	0	23.5

RN				
*Measure/edge type*	*Red*	*Green*	*Blue*	*Black*
True network (#)	10	4	1	2
True network (%)	58.8	23.5	5.88	11.8
Leaves (#)	2	2	1	2
Leaves (% E_L_)	28.57	28.57	14.29	28.57
Leaves (% N_et_)	11.76	11.76	5.88	11.76
In-hubs (#)	5	1	0	0
In-hubs(% E_L_)	83.33	16.67	0	0
In-hubs(% N_et_)	29.4	5.88	0	0

Leaves (% E_L_) refers to the percentage of leaf edges of a certain colour with respect to the total number of leaf edges, and leaves (% N_et_) refers to the percentage of leaf edges of a certain colour with respect to the entire network. Correspondingly for in-hubs. (In-hubs (%E_L_) refers to the percentage of in-hub edges of a certain colour with respect to the total number of in-hub edges, and In-hubs (%N_et_) refers to the percentage of in-hub edges of a certain colour with respect to the entire network.)

**Figure 4:**
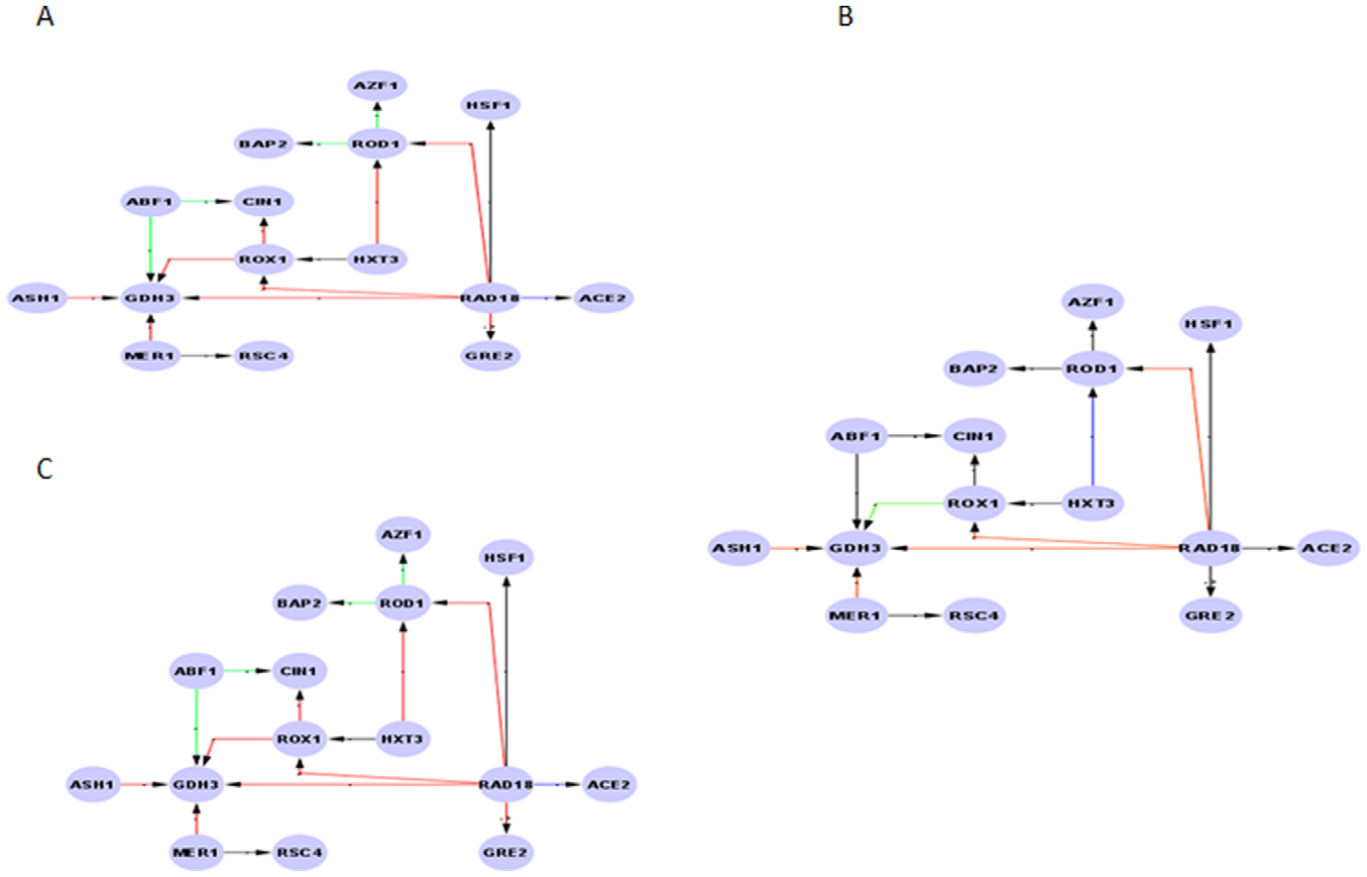
Visualisation of the results for FTCCT (A), FICPT (B) and RN (C) for an ensemble of size 24 containing 15 nodes. The colour of each edge reflects its mean TPR. Specifically, for black edges, 1≥TPR>0.75; for blue edges, 0.75≥TPR>0.5; for green edges, 0.5≥TPR>0.25; and for red edges, 0.25≥TPR>0.0.

For the second ensemble evaluation, we show a visualisation of the mean TPR of edges in the true network for FTCCT (Fig. 4A), FICPT (Fig. 4B) and RN (Fig. 4C). The edges are colour coded corresponding to their mean TPR. Specifically, for black edges, 1≥TPR>0.75; for blue edges, 0.75≥TPR>0.5; for green edges, 0.5≥TPR>0.25; and for red edges, 0,25≥TPR>0. Two items in the graph, *in-hubs* and *leaves* merit further analysis. An in-hub is a gene that has more than two incoming edges, and a leaf is a terminal gene that has exactly one incoming edge. Table 4 provides a quantitative summary of these qualitative visualisations. Table 4 was calculated by counting the number of leaf edges (leaf#) and in-hub edges (in-hub#) with respect to the occurrence of red, green, blue and black edges (Emmert-Streib and Altay, 2010). One can observe in Table 4 that, in general, the black and red leaf edges are much more frequent than the blue and green leaf edges. Likewise for the in-hub edges red and black edges are much more frequent than blue and green edges. Table 4 suggests that, in general, the probability for black edges (FTCCT = 58.8% and FICPT = 58.8%) is greater than that of red edges (FTCCT = 35.5% and FICPT = 29.4%) while RN shows a higher probability of red edges (58.8%) than the other types of edges (black edges (11.8%), green edges (23.5%) and blue edges (5.88%)) with respect to the entire network. For leaf nodes, the probability of black leaf edges was 85.7% for both FTCCT and FICPT, but that of red leaf edges was only 14.3%. For RN, this situation is reversed. In-hub edges show almost the same systematic bias for FTCCT and FICPT as shown by leaf edges, with a tendency towards black edges rather than red edges. Again for RN, the situation is reversed. This finding implies a systematic bias for FTCCT and FICPT towards better inference of the true network compared to RN.

### Reliability evaluation

#### Benchmark for reliability evaluation #4: Are FTCCT and FICPT more reliable than the single reverse-engineering methods

**Figure 5:**
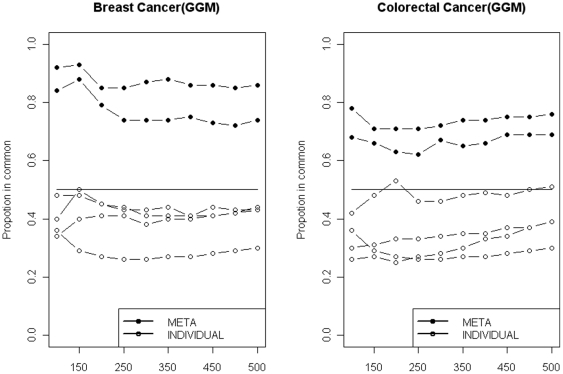
CAT plots of the breast cancer (left panel) and colorectal cancer (right panel) data. META represents FTCCT and FICPT, and INDIVIDUAL represents ARACNE, CLR, MRNET and RN. The black solid horizontal line indicates 50% agreement. The horizontal axis shows the size of the list, which is the number of genes. The line with solid circles represents META, and the line with open circles represents INDIVIDUAL.

Breast cancer data: Three breast cancer expression datasets have been used to analyse the reliability and reproducibility of ARACNe, CLR, MRNET, BN, RN, FTCCT and FICPT. We used CAT plots for reliability measurements. We followed the method introduced by [36] to produce CAT plots with one exception; we replace differentially expressed genes with a hub of the gene network. A master gene connected to other genes to form a hub. Genes connected to the master gene, excluding the master gene itself are ranked at the end of the process. Briefly, we chose the *BRCA1* gene as the master gene and took its hub as a benchmark study. For the three cancer datasets, three ranked lists that contain the *BRCA1* hub are produced. The first step in producing CAT plots for the biological network is to compare the top *n* genes identified in ranked list 1 and 2, treating the top *n* genes identified in the third dataset as a reference. Subsequently, CAT plots for the single reverse-engineering methods can be produced. For meta-analysis, two rather than three ranked lists are produced; the first ranked list is generated by applying meta-analysis approaches (FICPT and FTCCT) to the first and third datasets. Subsequently, the meta-analysis approaches are applied to the second and third datasets to produce the second ranked list. Lastly, the ranked lists are compared to produce CAT plots for meta-analysis. The CAT plots show the percentage in common among the top genes that have significant associations with *BRCA1*. Because, we would expect a sizeable number of gene interactions, the CAT plots are drawn for the top 500 genes, as shown in Fig. 5. In Fig. 5, ‘meta’ represents FTCCT and FICPT, and ‘individual’ represents ARACNe, CLR, MRNET, and RN. The CAT plot for BN was dropped because the datasets are too large to be inferred by Bayesian approaches. FTCCT and FICPT, in general, had higher reliability than the single reverse-engineering methods, suggesting that the results are more likely to be reproduced by an independent study. For example, the rediscovery rate was above 60% for FTCCT and FICPT, while it was below 50% for ARACNe, CLR, MRNET and RN. These rates are consistent with the simulation study, in which FTCCT and FICPT yielded more robust gene interactions based on ensemble evaluations, leading to both higher reproducibility and increased specificity.

Colorectal cancer data: Three colorectal cancer datasets were downloaded from a public repository (NBI). The CAT plots for both the single reverse-engineering methods and the meta-analysis approaches were produced by the same procedures as when *BRCA1* was the hub. Consistent with previous CAT plot results from the breast cancer data, Fig. 5 confirmed the higher re-discovery rate for FTCCT and FICPT compared to ARACNe, CLR, MRNET and RN.

## Discussion

Our results show that in practice, meta-analysis approaches perform better than individual reverse-engineering methods and drastically improve the accuracy and reliability of predictions from noisy datasets, as shown in simulated gene network and experimental datasets. The meta-analysis approaches in this paper were used to construct an ensemble of consistent and good-scoring networks, in contrast to the results of individual reverse-engineering methods. The reconstruction of a gene network by individual reverse-engineering methods often aims to reliably find the best-scoring network. Compared to generating gene networks from individual reverse-engineering methods, the complexity of meta-analysis approaches is negligible. It means that the meta-analysis approaches entail little additional complexity and they are not nearly as complex as the individual reverse-engineering methods. Thus, scalability to larger networks is possible using meta-analysis approaches. In this paper, we only considered small networks, and we plan to study the performance of meta-analysis on different network sizes in the future. The meta-analysis approach holds promise for improving the accuracy of any reverse-engineering method than can produce sufficiently diverse network predictions.

### Performance evaluations

Overall, meta-analysis approaches significantly outperform single reverse-engineering methods. In this paper, we accumulated inconsistent scoring networks generated by RN from multiple datasets to be integrated by meta-analysis approaches to produce more consistent and better-scoring networks. However, integrating datasets using conventional methods, such as copula-transform, to increase the AUC seems to have been ineffective at increasing the performance of any of the reverse-engineering methods. This finding indicates that using copula-transform may generate varying AUCs for any reverse-engineering method across datasets but neither significantly increases nor decreases their performance owing to data normalisation issues and a lack of temporal relationships among the datasets. Moreover, the median AUCs of both meta-analysis approaches outperformed BNs, suggesting that FTCCT and FICPT can increase the performance of individual inferring reverse-engineering methods on par with BNs, or even higher. This performance improvement occurs because meta-analysis approaches provide a systematic integrating framework compared to the copula-transform technique. As shown in previous results, significantly better AUCs for both FTCCT and FICPT compared to ARACNe, CLR, MRNET and RN indicate the usefulness of these meta-analysis approaches in increasing the number of true associations detected across datasets.

The tIDRs of FTCCT and FICPT were somewhat higher than the tIRRs of ARACNe, CLR, MRNET and RN. In the other words, we found that FTCCT and FICPT produce higher rates of true associations compared to ARACNe, CLR, MRNET and RN. The discovery rates also indicate that FTCCT and FICPT are more robust than ARACNe, CLR, MRNET and RN (excluding BN) and demonstrate that FTCCT and FICPT could somewhat overcome some problems suffered by individual reverse-engineering methods. As shown, we used the most stringent threshold for producing tIDR and tIRR (1e-12), yet both meta-analysis approaches still uncovered more true associations than any of the individual reverse-engineering methods.

We also examined the results at the level of individual edges to gain insight into how FCCT and FICPT can increase the performance of RN with ensemble evaluations. From simulation studies, one can see that the AUC of a single RN is not homogeneous across datasets but varies considerably across the whole network. One can see the effects of this inconsistency on network predictions in that a single RN can infer motif type 3 well but is less effective for motif types 1 and 2 across datasets. The inconsistency can also be observed where there are more red edges than black in the gene network inferred by RN. In contrast, FTCCT and FICPT diluted this inconsistency by effectively predicting all three motifs and increasing the proportion of black edges in the gene network. Thus, the high performance of these meta-analysis approaches occurs because the approaches are not biased towards any particular motifs in biological networks. Incorporating more motifs that can be reliably inferred by any reverse-engineering method could subsequently increase the performance of these methods such that more black edges than red edges would be present in the gene network. However, motif type 4 has not been investigated.

### Reliability evaluations

Inconsistency in reconstructing gene networks could also be measured using CAT plots. CAT plots measure both the reliability and reproducibility metrics of reverse-engineering methods in inferring and predicting gene networks. In our case, reliability indicates consistency, and the more consistent the gene network, the more accurate the predictions. Poor reproducibility and reliability have been major concerns, discouraging some biologists from trusting the results of microarray experiments. CAT plot results reveal that FTCCT and FICPT produce more reliable and reproducible results than ARACNe, CLR, MRNET and RN. It appears that FTCCT and FICPT consistently have the highest reproducibility/specificity in real biological datasets, regardless of the scale of heterogeneity among the datasets. This result also suggests that the gene networks produced by FTCCT and FICPT are more robust against noise and other hidden variables that might be embedded in different biological samples and datasets.

In conclusion, meta-analysis approaches have an intuitive appeal, and the results from this paper show that they work well in practice. We hope that the encouraging results presented here will stimulate further research on this topic.

## Supporting Information

Supporting Information S1 Generation of simulated datasets and parameters used.
(DOC)

Supporting Information S2 Software used for comparison and their settings.
(DOC)

Supporting Information S3 AUCs show detailed statistical summaries for BN, RNCT, MRNET, CLR and ARACNe compared to FTCCT and FICPT.
(DOC)

Supporting Information S4 Global measures of tIDR-tIRR for RNCT, MRNET, CLR and ARACNe compared to FICPT and FTCCT.
(DOC)

Supporting Information S5 Local measures for RNCT, FICPT and FTCCT.
(DOC)
