# Supplementary material for: Investigating Meta-Approaches for Reconstructing Gene Networks in a Mammalian Cellular Context
Source: PLoS One. 2012 Jan 9;7(1):e28713. doi: 10.1371/journal.pone.0028713 (PMC3253778; doi:10.1371/journal.pone.0028713)
Supplement: Supporting Information S1 — Generation of simulated datasets and parameters used. (DOC) [file pone.0028713.s001.doc]

**Supporting Information S1**

**(Datasets)**

**Simulation**

In total, we generated 24 different datasets by varying their biological and experimental noise settings with 1000 samples per dataset. To generate the first dataset, download SynTReN from the SynTReN site (<http://homes.esat.kuleuven.be/~kmarchal/SynTReN/index.html>). Then, extract the software onto your desktop. In the generated SynTReN directory there should be a batch file called *syntren_gui.bat*; double-click on the batch file to run SynTReN. The graphical interface will appear. In the graphical interface, there are 15 default settings; the first setting is *Burin period* and the last is *Random seed*. To generate 1000 samples, modify the default value of *Nr samples per experiment* setting from 1 to 10 samples per experiment and leave the *Nr experiment* setting at its default value. Next, to make a network of 15 genes, change the value of the *Nr nodes* setting from 100 to 15. To approximate a mammalian network structure, we chose a yeast network by changing the *Source network* setting from its default value to *Yeast* (*Saccharomyces cerevisiae*). The yeast network is stored in the *sourceNetwork* sub-directory, which can be reached by navigating from the SynTReN main directory, and is labelled *Yeast_full_nn* under the *SIF* file. Another file contains a yeast network labelled *Yeast_full*, but, this network is unsuitable for inferring gene networks because it contains a feedback loop. SynTReN can generate biological and experimental noise, each of our datasets contains different biological and experimental noise. To vary the biological and experimental noise, change the *Biological noise [0..1]* and *Experimental noise [0..1]* settings to new values. Our datasets were generated by grouping biological and experimental noises as shown in Table 1. Eventually, press the *Generate datasets (~1 minute)* button once to generate the first dataset. Typically, there are six files generated by SynTReN, and only one of these files is important: *nn10_nbgr100_hop0.3_bionoise0.1_expnoise0.1_corrnoise0.1_neighAdd_maxExpr1_dataset (*the name of the output file may vary). All 24 datasets can be generated by repeating these procedures and varying the noise. For every dataset, leave the *Nr experiments, Nr samples per experiment, Nr nodes, and Source Network* settings intact but vary the *Biological noises [0..1]* and *Experimental noises [0..1]* settings.

**Table 1.** Varying biological and experimental noises tests generated using SynTReN

| **Group** | **Datasets** | **Biological noise** | **Experimental noise** | **Species** |
| --- | --- | --- | --- | --- |
| 1. | 1 | 0.1 | 0.1 | Saccharomyces cerevisiae |
|  | 2 | 0.2 | 0.1 | Saccharomyces cerevisiae |
|  | 3 | 0.3 | 0.1 | Saccharomyces cerevisiae |
|  | 4 | 0.4 | 0.1 | Saccharomyces cerevisiae |
| 2. | 5 | 0.1 | 0.5 | Saccharomyces cerevisiae |
|  | 6 | 0.1 | 0.6 | Saccharomyces cerevisiae |
|  | 7 | 0.1 | 0.7 | Saccharomyces cerevisiae |
|  | 8 | 0.1 | 0.8 | Saccharomyces cerevisiae |
| 3. | 9 | 0.2 | 0.1 | Saccharomyces cerevisiae |
|  | 10 | 0.3 | 0.1 | Saccharomyces cerevisiae |
|  | 11 | 0.4 | 0.1 | Saccharomyces cerevisiae |
|  | 12 | 0.5 | 0.1 | Saccharomyces cerevisiae |
| 4. | 13 | 0.6 | 0.1 | Saccharomyces cerevisiae |
|  | 14 | 0.7 | 0.1 | Saccharomyces cerevisiae |
|  | 15 | 0.8 | 0.1 | Saccharomyces cerevisiae |
|  | 16 | 0.9 | 0.1 | Saccharomyces cerevisiae |
| 5. | 17 | 0.1 | 0.2 | Saccharomyces cerevisiae |
|  | 18 | 0.1 | 0.3 | Saccharomyces cerevisiae |
|  | 19 | 0.1 | 0.4 | Saccharomyces cerevisiae |
|  | 20 | 0.1 | 0.5 | Saccharomyces cerevisiae |
| 6. | 21 | 0.1 | 0.6 | Saccharomyces cerevisiae |
|  | 22 | 0.1 | 0.7 | Saccharomyces cerevisiae |
|  | 23 | 0.1 | 0.8 | Saccharomyces cerevisiae |
|  | 24 | 0.1 | 0.9 | Saccharomyces cerevisiae |

**Experimental data**

The performance of ARACNe, CLR, MRNET and RN and FTCCT and FICPT were also evaluated in real data settings based on two cancers: breast cancer and colorectal cancer. The complete colorectal cancer datasets are composed of three datasets obtained from a public repository (NCBI) with 62 samples in the first dataset (accession code GSE 12945), 55 samples in the second dataset (accession code GSE 17357) and 73 samples in the third dataset (accession code GSE 13067). The breast cancer expression profile consists of 266 samples in the first dataset (accession code GSE 21653), 51 samples in the second dataset (accession code GSE 17907) and 55 samples in the third dataset (accession code GSE 16391). All of the datasets were pre-processed using gcRMA in Bioconductor.
