# Supplementary material for: Investigating Meta-Approaches for Reconstructing Gene Networks in a Mammalian Cellular Context
Source: PLoS One. 2012 Jan 9;7(1):e28713. doi: 10.1371/journal.pone.0028713 (PMC3253778; doi:10.1371/journal.pone.0028713)
Supplement: Supporting Information S2 — Software used for comparison and their settings. (DOC) [file pone.0028713.s002.doc]

**Supporting Information S2**

**(Software settings)**

Here we describe step-by-step procedures for inferring the simulated gene regulatory network. For RNCT, we used the ParCorA software, which can be downloaded from (<http://mendes.vbi.vt.edu/tiki-index.php?page=Software>). The software is available in a Windows version in a zip file and can be extracted using any software compression tool. After extracting, there will be 10 files (8 application files, 2 dataset files and 1 readme file). To use the software for inferring, for example using Pearson correlation zero-order, create a new folder and copy an execution file named Pearson0th.exe and 1 dataset out of the 24 datasets. Rename the dataset *data.txt*; the .txt extension is necessary because all of the ParCorA software detects an input file with the name *data.txt*. Run the application by double-clicking on the execution file, and then a DOS interface will appear. There are two variables that must be established before the software can make an inference. The first variable ‘*How many variables?’* asks about the number of genes, and the second variable ‘*How many observations?*’ requests the number of samples in the dataset. After inputting the variables by pressing the *Enter* key, the software will generate 7 different files. The most important of these files is *Pearson0th_matrix.xls.* This file contains the result of inferring the dataset using Pearson correlation, which produces an adjacency matrix with entries that are the Pearson correlation coefficients. The gene names are placed along the top and the left sides of the adjacency matrix. Repeat these steps for each of the remaining 23 datasets. ParCorA can infer up to third-order inferences using either Pearson or Spearman correlation coefficients.

For ARACNe, CLR and MRNET, we utilised the *minet* package, which can be downloaded from (<http://cran.r-project.org/web/packages/minet/>) into the R environment. All three algorithms (ARACNe, CLR and MRNET) use the same procedure to infer a gene regulatory network, but they use different functions. To infer the first simulated dataset, transform the datasets into the *minet* format using the *build.mim* function. The function produces an adjacency matrix for the datasets in the form of mutual information. The adjacency matrix can then be fed into the *aracne* function, the *clr* function or the *mrnet* function. To infer using ARACNe, insert the adjacency matrix into the *aracne* function, and the function will output the inferred result in the form of mutual information. The same procedure is used for CLR and MRNET but using the *clr* and *mrnet* functions, respectively, for inferring. The output of these functions is mutual information.

To infer those datasets using BNs, we downloaded software from (<http://www.bioss.ac.uk/download/adriano>). First-time users must register by entering their email address into a form at the end of the website. The site contains software by two different authors under the software section: *Software from Adriano V. Werhli* and *Software from Marco Grzegorczyk*. We used *Software from Adriano V.Werhli* as our BNs software of choice. After extraction, the folder contains 10 matlab files and 2 sets of instructions for running BNs. For inferring using BNs, we utilised 5 matlab files *Compute_Prior_Info.mat*, *Score_all.mat, start_order_mcmc.mat*, *AUROC_observational.mat* and *TP_for_FP5_observational.mat*. To use these files first activate the matlab software by double-clicking on the matlab icon and load all five aforementioned matlab files into matlab’s working environment. Next, load one of the simulated datasets into matlab’s working environment and execute the Compute_Prior_Info function by typing the command Compute_Prior_Info(data) (the data parameter represents the dataset name) into matlab’s command line environment. The output of the function is then fed to the *Score_all* function by leaving all parameters at their default values and executed in the same way as the previous function. Eventually, an adjacency matrix can be generated by feeding the output of *Score_all* function to *start_order_mcmc*.
