# Supplementary material for: Investigating Meta-Approaches for Reconstructing Gene Networks in a Mammalian Cellular Context
Source: PLoS One. 2012 Jan 9;7(1):e28713. doi: 10.1371/journal.pone.0028713 (PMC3253778; doi:10.1371/journal.pone.0028713)
Supplement: Supporting Information S3 — AUCs show detailed statistical summaries for BN, RNCT, MRNET, CLR and ARACNe compared to FTCCT and FICPT. (DOC) [file pone.0028713.s003.doc]

**Supporting Information S3**

**(Global measures-AUC)**

BN AUC

| Scale-free network |
| --- |
| | **Dataset** | **BN** | **MFT** | | --- | --- | --- | | 2 | 0.8046 | 0.8331 | | 3 | 0.7952667 | 0.8251 | | 4 | 0.80665 | 0.8232 | | 5 | 0.79864 | 0.8195 | | 6 | 0.7882167 | 0.8177 | | 7 | 0.7855143 | 0.8158 | | 8 | 0.7848 | 0.8121 | | 9 | 0.7893778 | 0.8158 | | 10 | 0.78842 | 0.8115 | | 11 | 0.7880545 | 0.8134 | | 12 | 0.785825 | 0.8041 | | 13 | 0.778823 | 0.8041 | | 14 | 0.7745571 | 0.8029 | | 15 | 0.7727333 | 0.8029 | | 16 | 0.764475 | 0.7974 | | 17 | 0.7639118 | 0.7983 | | 18 | 0.7621944 | 0.7955 | | 19 | 0.7614368 | 0.7909 | | 20 | 0.76109 | 0.7884 | | 21 | 0.6566714 | 0.7884 | | 22 | 0.6595955 | 0.785 | | 23 | 0.6597739 | 0.7801 | | 24 | 0.6611833 | 0.7789 | | **p-value** |  | 9.424e-07 | |
|  |

ARACNe AUC

| Scale-free network |
| --- |
| | **Dataset** | **ARACNe** | **MFT** | | --- | --- | --- | | 2 | 0.7186 | 0.8331 | | 3 | 0.7037 | 0.8251 | | 4 | 0.6871 | 0.8232 | | 5 | 0.6846 | 0.8195 | | 6 | 0.6762 | 0.8177 | | 7 | 0.6696 | 0.8158 | | 8 | 0.6613 | 0.8121 | | 9 | 0.6551 | 0.8158 | | 10 | 0.6492 | 0.8115 | | 11 | 0.642 | 0.8134 | | 12 | 0.6327 | 0.8041 | | 13 | 0.629 | 0.8041 | | 14 | 0.6215 | 0.8029 | | 15 | 0.6188 | 0.8029 | | 16 | 0.6158 | 0.7974 | | 17 | 0.6151 | 0.7983 | | 18 | 0.6141 | 0.7955 | | 19 | 0.6118 | 0.7909 | | 20 | 0.6077 | 0.7884 | | 21 | 0.6066 | 0.7884 | | 22 | 0.6029 | 0.785 | | 23 | 0.6019 | 0.7801 | | 24 | 0.6006 | 0.7789 | | **p-value** |  | 6.609e-09 | |
|  |

MRNET AUC

| Scale-free network |
| --- |
| | **Dataset** | **MRNET** | **MFT** | | --- | --- | --- | | 2 | 0.7113 | 0.8331 | | 3 | 0.6824 | 0.8251 | | 4 | 0.659 | 0.8232 | | 5 | 0.6577 | 0.8195 | | 6 | 0.5548 | 0.8177 | | 7 | 0.5707 | 0.8158 | | 8 | 0.5821 | 0.8121 | | 9 | 0.5911 | 0.8158 | | 10 | 0.5955 | 0.8115 | | 11 | 0.5837 | 0.8134 | | 12 | 0.5745 | 0.8041 | | 13 | 0.5714 | 0.8041 | | 14 | 0.5645 | 0.8029 | | 15 | 0.5669 | 0.8029 | | 16 | 0.5696 | 0.7974 | | 17 | 0.5751 | 0.7983 | | 18 | 0.5784 | 0.7955 | | 19 | 0.5725 | 0.7909 | | 20 | 0.5675 | 0.7884 | | 21 | 0.5659 | 0.7884 | | 22 | 0.5618 | 0.785 | | 23 | 0.5635 | 0.7801 | | 24 | 0.5654 | 0.7789 | | **p-value** |  | 3.591e-08 | |
|  |
|  |

CLR AUC

| Scale-free network |
| --- |
| | **Dataset** | **MRNET** | **MFT** | | --- | --- | --- | | 2 | 0.7592 | 0.8331 | | 3 | 0.7307 | 0.8251 | | 4 | 0.7053 | 0.8232 | | 5 | 0.6994 | 0.8195 | | 6 | 0.6977 | 0.8177 | | 7 | 0.6964 | 0.8158 | | 8 | 0.6875 | 0.8121 | | 9 | 0.6925 | 0.8158 | | 10 | 0.6944 | 0.8115 | | 11 | 0.6866 | 0.8134 | | 12 | 0.6795 | 0.8041 | | 13 | 0.6756 | 0.8041 | | 14 | 0.6703 | 0.8029 | | 15 | 0.667 | 0.8029 | | 16 | 0.6655 | 0.7974 | | 17 | 0.6694 | 0.7983 | | 18 | 0.6718 | 0.7955 | | 19 | 0.6685 | 0.7909 | | 20 | 0.6651 | 0.7884 | | 21 | 0.6633 | 0.7884 | | 22 | 0.6605 | 0.785 | | 23 | 0.6589 | 0.7801 | | 24 | 0.6582 | 0.7789 | | **p-value** |  | 3.591e-08 | |
|  |

RNCT AUC

| Scale-free network |
| --- |
| | **Dataset** | **MRNET** | **MFT** | | --- | --- | --- | | 2 | 0.7484 | 0.8331 | | 3 | 0.7459 | 0.8251 | | 4 | 0.7431 | 0.8232 | | 5 | 0.741 | 0.8195 | | 6 | 0.7399 | 0.8177 | | 7 | 0.7376 | 0.8158 | | 8 | 0.7351 | 0.8121 | | 9 | 0.7344 | 0.8158 | | 10 | 0.7306 | 0.8115 | | 11 | 0.726 | 0.8134 | | 12 | 0.7217 | 0.8041 | | 13 | 0.7174 | 0.8041 | | 14 | 0.7134 | 0.8029 | | 15 | 0.7094 | 0.8029 | | 16 | 0.7055 | 0.7974 | | 17 | 0.7069 | 0.7983 | | 18 | 0.7063 | 0.7955 | | 19 | 0.7049 | 0.7909 | | 20 | 0.7034 | 0.7884 | | 21 | 0.7017 | 0.7884 | | 22 | 0.6998 | 0.785 | | 23 | 0.6978 | 0.7801 | | 24 | 0.6957 | 0.7789 | | **p-value** |  | 6.609e-09 | |
|  |
