# Supplementary material for: Investigating Meta-Approaches for Reconstructing Gene Networks in a Mammalian Cellular Context
Source: PLoS One. 2012 Jan 9;7(1):e28713. doi: 10.1371/journal.pone.0028713 (PMC3253778; doi:10.1371/journal.pone.0028713)
Supplement: Supporting Information S4 — Global measures of tIDR-tIRR for RNCT, MRNET, CLR and ARACNe compared to FICPT and FTCCT. (DOC) [file pone.0028713.s004.doc]

**Supporting Information S4**

**(Global measure: tIDR-tIRR)**

RNCT tIDR

| Scale-free network |
| --- |
| | **Dataset** | **RNCT-tIRR** | **MFT-tIDR** | | --- | --- | --- | | 2 | 0 | 4 | | 3 | 0 | 4 | | 4 | 0 | 4 | | 5 | 0 | 4 | | 6 | 0 | 6 | | 7 | 0 | 6 | | 8 | 0 | 6 | | 9 | 0 | 6 | | 10 | 0 | 4 | | 11 | 0 | 4 | | 12 | 0 | 6 | | 13 | 4 | 6 | | 14 | 4 | 6 | | 15 | 4 | 6 | | 16 | 4 | 6 | | 17 | 4 | 8 | | 18 | 4 | 8 | | 19 | 6 | 10 | | 20 | 6 | 10 | | 21 | 6 | 10 | | 22 | 6 | 10 | | 23 | 6 | 10 | | 24 | 6 | 10 | | **p-value** |  | 3.122e-05 | |
|  |

ARACNe tIDR

| Scale-free network |
| --- |
| | **Dataset** | **Aracne-tIRR** | **MFT-tIDR** | | --- | --- | --- | | 2 | 0 | 14 | | 3 | 0 | 14 | | 4 | 0 | 14 | | 5 | 0 | 14 | | 6 | 0 | 16 | | 7 | 0 | 16 | | 8 | 0 | 16 | | 9 | 0 | 16 | | 10 | 0 | 14 | | 11 | 2 | 14 | | 12 | 2 | 16 | | 13 | 2 | 16 | | 14 | 2 | 16 | | 15 | 2 | 16 | | 16 | 2 | 16 | | 17 | 2 | 18 | | 18 | 2 | 18 | | 19 | 2 | 20 | | 20 | 2 | 20 | | 21 | 2 | 20 | | 22 | 2 | 20 | | 23 | 2 | 20 | | 24 | 2 | 20 | | **p-value** |  | 2.817e-09 | |
|  |

MRNET tIDR

| Scale-free network |
| --- |
| | **Dataset** | **Mrnet-tIRR** | **MFT-tIDR** | | --- | --- | --- | | 2 | 0 | 14 | | 3 | 0 | 14 | | 4 | 0 | 14 | | 5 | 0 | 14 | | 6 | 0 | 16 | | 7 | 0 | 16 | | 8 | 0 | 16 | | 9 | 0 | 16 | | 10 | 0 | 14 | | 11 | 2 | 18 | | 12 | 2 | 18 | | 13 | 2 | 18 | | 14 | 2 | 20 | | 15 | 2 | 18 | | 16 | 2 | 18 | | 17 | 2 | 20 | | 18 | 2 | 20 | | 19 | 2 | 20 | | 20 | 2 | 20 | | 21 | 2 | 20 | | 22 | 2 | 20 | | 23 | 2 | 20 | | 24 | 2 | 20 | | **p-value** |  | 2.887e-09 | |
|  |

CLR tIDR

| Scale-free network |
| --- |
| | **Dataset** | **CLR-tIRR** | **MFT-tIDR** | | --- | --- | --- | | 2 | 0 | 14 | | 3 | 0 | 14 | | 4 | 0 | 14 | | 5 | 0 | 14 | | 6 | 0 | 16 | | 7 | 0 | 16 | | 8 | 0 | 16 | | 9 | 0 | 16 | | 10 | 0 | 14 | | 11 | 2 | 14 | | 12 | 2 | 16 | | 13 | 2 | 16 | | 14 | 2 | 16 | | 15 | 2 | 16 | | 16 | 2 | 16 | | 17 | 2 | 18 | | 18 | 2 | 18 | | 19 | 2 | 20 | | 20 | 2 | 20 | | 21 | 2 | 20 | | 22 | 14 | 20 | | 23 | 14 | 20 | | 24 | 14 | 20 | | **p-value** |  | 1.119e-08 | |
|  |
