# Supplementary material for: Investigating Meta-Approaches for Reconstructing Gene Networks in a Mammalian Cellular Context
Source: PLoS One. 2012 Jan 9;7(1):e28713. doi: 10.1371/journal.pone.0028713 (PMC3253778; doi:10.1371/journal.pone.0028713)
Supplement: Supporting Information S5 — Local measures for RNCT, FICPT and FTCCT. (DOC) [file pone.0028713.s005.doc]

**Supporting Information S5**

**(Local measures)**

Total motif in network

| Chain motif |
| --- |
| | 1. | RAD18 -> ROD1 -> BAP2 | | --- | --- | | 2. | RAD18 -> ROD1 -> AZF1 | | 3. | HXT3 -> ROD1 -> AZF1 | | 4. | HXT3 -> ROX1 -> GDH3 | | 5. | RAD18 -> ROX1 -> CIN1 | |
| Collider motif |
| | 1. | RAD18 -> ROD1 <- HXT3 | | --- | --- | | 2. | HXT3 -> ROX1 <- RAD18 | | 3. | ROX1 -> GDH3 <- ABH1 | | 4. | ROX1 -> GDH3 <- ASH1 | | 5. | ROX1 -> GDH3 <- MER1 | | 6. | ROX1 -> GDH3 <- RAD18 | | 7. | ABF1 -> GDH3 <- ASH1 | | 8. | ABF1 -> GDH3 <- MER1 | | 9 | ABF1 -> GDH3 <- RAD18 | | 10. | ROX1 -> CIN1 <- ABF1 | |
| Fork motif |
| | 1. | BAP2 <- ROD1 ->AZF1 | | --- | --- | | 2. | ROD1 <- HXT3 -> ROX1 | | 3. | CIN1 <- ROX1 -> GDH3 | | 4. | ROD1 <- RAD18 -> GDH3 | | 5. | ROD1 <- RAD18 -> ROX1 | | 6. | ACE2 <- RAD18 -> ROX1 | | 7. | ACE2 <- RAD18 -> GDH3 | | 8. | RSC4 <- MER1 -> GDH3 | | 9. | CIN1 <- ABF1 -> GDH3 | | 10. | HSF1 <- RAD18 -> ACE2 | | 11. | HSF1 <- RAD18 -> ROD1 | | 12. | HSF2 <- RAD18 -> ROX1 | | 13. | HSF2 <- RAD18 -> GDH3 | | 14. | ACE2 <- RAD18 -> GRE2 | | 15. | ACE2 <- RAD18 -> ROD1 | | 16. | GRE2 <- RAD18 -> ROD1 | | 17. | GRE2 <- RAD18 -> ROX1 | | 18. | GRE2 <- RAD18 -> GDH3 | |

MOTIF FTCCT

| Chain motif |
| --- |
| |  | ***Motif*** | ***TPR (AB)*** | ***TPR(BC)*** | ***TNR(AC)*** | ***p*** | | --- | --- | --- | --- | --- | --- | | 1. | RAD18 -> ROD1 -> BAP2 | 0 | 1 | 0.5652174 | 0.5217391 | | 2. | RAD18 -> ROD1 -> AZF1 | 0 | 1 | 1 | 0.6666667 | | 3. | HXT3 -> ROD1 -> AZF1 | 0.9130435 | 1 | 1 | 0.9710145 | | 4. | HXT3 -> ROX1 -> GDH3 | 1 | 0.9130435 | 1 | 0.9710145 | | 5. | RAD18 -> ROX1 -> CIN1 | 0 | 0 | 1 | 0.3333333 | |  |  |  |  | ***Mean(p)*** | 0.6927536 | |
| Collider motif |
| |  | ***Motif*** | ***TPR (AB)*** | ***TPR(BC)*** | ***TNR(AC)*** | ***p*** | | --- | --- | --- | --- | --- | --- | | 1. | RAD18 -> ROD1 <- HXT3 | 0 | 0.9130435 | 1 | 0.6376812 | | 2. | HXT3 -> ROX1 <- RAD18 | 1 | 0 | 1 | 0.6666667 | | 3. | ROX1 -> GDH3 <- ABF1 | 0.9130435 | 1 | 1 | 0.9710145 | | 4. | ROX1 -> GDH3 <- ASH1 | 0.9130435 | 0 | 1 | 0.6376812 | | 5. | ROX1 -> GDH3 <- MER1 | 0.9130435 | 0 | 1 | 0.6376812 | | 6. | ROX1 -> GDH3 <- RAD18 | 0.9130435 | 0 | 0 | 0.3043478 | | 7. | ABF1 -> GDH3 <- ASH1 | 1 | 0 | 1 | 0.6666667 | | 8. | ABF1 -> GDH3 <- MER1 | 1 | 0 | 1 | 0.6666667 | | 9 | ABF1 -> GDH3 <- RAD18 | 1 | 0 | 1 | 0.6666667 | | 10. | ROX1 -> CIN1 <- ABF1 | 0.4782609 | 1 | 1 | 0.826087 | |  |  |  |  | ***Mean(p)*** | 0.668116 | |
| Fork motif |
| |  | ***Motif*** | ***TPR (AB)*** | ***TPR(BC)*** | ***TNR(AC)*** | ***p*** | | --- | --- | --- | --- | --- | --- | | 1. | BAP2 <- ROD1 ->AZF1 | 1 | 1 | 0.1739130 | 0.7246377 | | 2. | ROD1 <- HXT3 -> ROX1 | 0.9130435 | 1 | 0 | 0.6376812 | | 3. | CIN1 <- ROX1 -> GDH3 | 0.4782609 | 0.9130435 | 1 | 0.7971015 | | 4. | ROD1 <- RAD18 -> GDH3 | 0 | 0 | 1 | 0.3333333 | | 5. | ROD1 <- RAD18 -> ROX1 | 0 | 0 | 0 | 0 | | 6. | ACE2 <- RAD18 -> ROX1 | 1 | 0 | 0.6956522 | 0.5652174 | | 7. | ACE2 <- RAD18 -> GDH3 | 1 | 0 | 0.1304348 | 0.3768116 | | 8. | RSC4 <- MER1 -> GDH3 | 1 | 0 | 0.9565217 | 0.6521739 | | 9. | CIN1 <- ABF1 -> GDH3 | 1 | 1 | 1 | 1 | | 10. | HSF1 <- RAD18 -> ACE2 | 1 | 1 | 0.2173913 | 0.7391304 | | 11. | HSF1 <- RAD18 -> ROD1 | 1 | 0 | 1 | 0.6666667 | | 12. | HSF1 <- RAD18 -> ROX1 | 1 | 0 | 1 | 0.6666667 | | 13. | HSF1 <- RAD18 -> GDH3 | 1 | 0 | 1 | 0.6666667 | | 14. | ACE2 <- RAD18 -> GRE2 | 1 | 1 | 0 | 0.6666667 | | 15. | ACE2 <- RAD18 -> ROD1 | 1 | 0 | 0 | 0.3333333 | | 16. | GRE2 <- RAD18 -> ROD1 | 1 | 0 | 0.04347826 | 0.3478261 | | 17. | GRE2 <- RAD18 -> ROX1 | 1 | 0 | 0.3478261 | 0.4492754 | | 18. | GRE2 <- RAD18 -> GDH3 | 1 | 0 | 1 | 0.6666667 | |  |  |  |  | ***Mean(p)*** | 0.5716586 | |

MOTIF FICPT

| Chain motif |
| --- |
| |  | ***Motif*** | ***TPR (AB)*** | ***TPR(BC)*** | ***TPR(AC)*** | ***p*** | | --- | --- | --- | --- | --- | --- | | 1. | RAD18 -> ROD1 -> BAP2 | 0 | 1 | 1 | 0.6666667 | | 2. | RAD18 -> ROD1 -> AZF1 | 0 | 1 | 1 | 0.6666667 | | 3. | HXT3 -> ROD1 -> AZF1 | 0.6956522 | 1 | 1 | 0.8985507 | | 4. | HXT3 -> ROX1 -> GDH3 | 0.8695652 | 0.3478261 | 1 | 0.7391304 | | 5. | RAD18 -> ROX1 -> CIN1 | 0 | 1 | 1 | 0.6666667 | |  |  |  |  | ***Mean(p)*** | 0.7275362 | |
| Collider motif |
| |  | ***Motif*** | ***TPR (AB)*** | ***TPR(BC)*** | ***TPR(AC)*** | ***p*** | | --- | --- | --- | --- | --- | --- | | 1. | RAD18 -> ROD1 <- HXT3 | 0 | 0.6956522 | 0 | 0.2318841 | | 2. | HXT3 -> ROX1 <- RAD18 | 0.8695652 | 0 | 0 | 0.2898551 | | 3. | ROX1 -> GDH3 <- ABF1 | 0.3478261 | 1 | 1 | 0.7826087 | | 4. | ROX1 -> GDH3 <- ASH1 | 0.3478261 | 0 | 1 | 0.4492754 | | 5. | ROX1 -> GDH3 <- MER1 | 0.3478261 | 0 | 1 | 0.4492754 | | 6. | ROX1 -> GDH3 <- RAD18 | 0.3478261 | 0 | 0 | 0.1159420 | | 7. | ABF1 -> GDH3 <- ASH1 | 1 | 0 | 1 | 0.6666667 | | 8. | ABF1 -> GDH3 <- MER1 | 1 | 0 | 1 | 0.6666667 | | 9 | ABF1 -> GDH3 <- RAD18 | 1 | 0 | 1 | 0.6666667 | | 10. | ROX1 -> CIN1 <- ABF1 | 1 | 1 | 1 | 1 | |  |  |  |  | ***Mean(p)*** | 0.5318841 | |
| Fork motif |
| |  | ***Motif*** | ***TPR (AB)*** | ***TPR(BC)*** | ***TPR(AC)*** | ***p*** |  | | | --- | --- | --- | --- | --- | --- | --- | --- | | 1. | BAP2 <- ROD1 ->AZF1 | 1 | 1 | 0.1739130 | 0.1739130 |  | | | 2. | ROD1 <- HXT3 -> ROX1 | 0.6956522 | 0.8695652 | 0.3043478 | 0.6956522 | 0.8695652 | 0.3043478 | | 3. | CIN1 <- ROX1 -> GDH3 | 1 | 0.3478261 | 1 | 1 | 0.3478261 | 1 | | 4. | ROD1 <- RAD18 -> GDH3 | 0 | 0 | 1 | 0.3333333 | 0.3478261 | 1 | | 5. | ROD1 <- RAD18 -> ROX1 | 0 | 0 | 0.3043478 | 0.1014493 |  | | | 6. | ACE2 <- RAD18 -> ROX1 | 1 | 0 | 1 | 0.6666667 |  | | | 7. | ACE2 <- RAD18 -> GDH3 | 1 | 0 | 0.3043478 | 0.4347826 |  | | | 8. | RSC4 <- MER1 -> GDH3 | 1 | 0 | 0.9130435 | 0.6376812 |  | | | 9. | CIN1 <- ABF1 -> GDH3 | 0 | 1 | 1 | 0.6666667 |  | | | 10. | HSF1 <- RAD18 -> ACE2 | 1 | 1 | 0.1304348 | 0.710145 |  | | | 11. | HSF1 <- RAD18 -> ROD1 | 1 | 0 | 1 | 0.6666667 |  | | | 12. | HSF1 <- RAD18 -> ROX1 | 1 | 0 | 1 | 0.6666667 |  | | | 13. | HSF1 <- RAD18 -> GDH3 | 1 | 0 | 1 | 0.6666667 |  | | | 14. | ACE2 <- RAD18 -> GRE2 | 1 | 1 | 0 | 0.6666667 |  | | | 15. | ACE2 <- RAD18 -> ROD1 | 1 | 0 | 0 | 0.6666667 |  | | | 16. | GRE2 <- RAD18 -> ROD1 | 1 | 0 | 0.2173913 | 0.4057971 |  | | | 17. | GRE2 <- RAD18 -> ROX1 | 1 | 0 | 0.6521739 | 0.5507246 |  | | | 18. | GRE2 <- RAD18 -> GDH3 | 1 | 0 | 1 | 0.6666667 |  | | |  |  |  |  | ***Mean(p)*** | 0.5764896 |  | | |

MOTIF RNCCT

| Chain motif |
| --- |
| |  | ***Motif*** | ***TPR (AB)*** | ***TPR(BC)*** | ***TPR(AC)*** | ***p*** | | --- | --- | --- | --- | --- | --- | | 1. | RAD18 -> ROD1 -> BAP2 | 0 | 0.3043478 | 1 | 0.4347826 | | 2. | RAD18 -> ROD1 -> AZF1 | 0 | 0.3478261 | 1 | 0.4347826 | | 3. | HXT3 -> ROD1 -> AZF1 | 0 | 0.3478261 | 1 | 0.4347826 | | 4. | HXT3 -> ROX1 -> GDH3 | 0 | 0 | 1 | 0.3333333 | | 5. | RAD18 -> ROX1 -> CIN1 | 0 | 0 | 1 | 0.3333333 | |  |  |  |  | ***Mean(p)*** | 0.3942029 | |
| Collider motif |
| |  | ***Motif*** | ***TPR (AB)*** | ***TPR(BC)*** | ***TPR(AC)*** | ***p*** | | --- | --- | --- | --- | --- | --- | | 1. | RAD18 -> ROD1 <- HXT3 | 0 | 0 | 0 | 0 | | 2. | HXT3 -> ROX1 <- RAD18 | 0 | 0 | 0 | 0 | | 3. | ROX1 -> GDH3 <- ABF1 | 0 | 0.08695652 | 1 | 0.3623188 | | 4. | ROX1 -> GDH3 <- ASH1 | 0 | 0 | 1 | 0.3333333 | | 5. | ROX1 -> GDH3 <- MER1 | 0 | 0 | 1 | 0.3333333 | | 6. | ROX1 -> GDH3 <- RAD18 | 0 | 0 | 0 | 0 | | 7. | ABF1 -> GDH3 <- ASH1 | 0.08695652 | 0 | 1 | 0.3623188 | | 8. | ABF1 -> GDH3 <- MER1 | 0.08695652 | 0 | 1 | 0.3623188 | | 9 | ABF1 -> GDH3 <- RAD18 | 0.08695652 | 0 | 1 | 0.3623188 | | 10. | ROX1 -> CIN1 <- ABF1 | 0 | 0.2608696 | 1 | 0.4202899 | |  |  |  |  | ***Total*** | 0.2536232 | |
| Fork motif |
| |  | ***Motif*** | ***TPR (AB)*** | ***TPR(BC)*** | ***TPR(AC)*** | ***p*** | | --- | --- | --- | --- | --- | --- | | 1. | BAP2 <- ROD1 ->AZF1 | 0.3043478 | 0.3478261 | 1 | 0.5507246 | | 2. | ROD1 <- HXT3 -> ROX1 | 0 | 0 | 1 | 0.3333333 | | 3. | CIN1 <- ROX1 -> GDH3 | 0 | 0 | 1 | 0.3333333 | | 4. | ROD1 <- RAD18 -> GDH3 | 0 | 0 | 1 | 0.3333333 | | 5. | ROD1 <- RAD18 -> ROX1 | 0 | 0 | 1 | 0.3333333 | | 6. | ACE2 <- RAD18 -> ROX1 | 0.5652174 | 0 | 1 | 0.5217391 | | 7. | ACE2 <- RAD18 -> GDH3 | 0.5652174 | 0 | 1 | 0.5217391 | | 8. | RSC4 <- MER1 -> GDH3 | 1 | 0 | 1 | 0.6666667 | | 9. | CIN1 <- ABF1 -> GDH3 | 0.2608696 | 0.08695652 | 1 | 0.4492754 | | 10. | HSF1 <- RAD18 -> ACE2 | 1 | 0.5652174 | 1 | 0.8550725 | | 11. | HSF1 <- RAD18 -> ROD1 | 1 | 0 | 1 | 0.6666667 | | 12. | HSF1 <- RAD18 -> ROX1 | 1 | 0 | 1 | 0.6666667 | | 13. | HSF1 <- RAD18 -> GDH3 | 1 | 0 | 1 | 0.6666667 | | 14. | ACE2 <- RAD18 -> GRE2 | 0.5652174 | 0.2173913 | 0.8695652 | 0.5507246 | | 15. | ACE2 <- RAD18 -> ROD1 | 0.5652174 | 0 | 1 | 0.5217391 | | 16. | GRE2 <- RAD18 -> ROD1 | 0.2173913 | 0 | 1 | 0.4057971 | | 17. | GRE2 <- RAD18 -> ROX1 | 0.2173913 | 0 | 1 | 0.4057971 | | 18. | GRE2 <- RAD18 -> GDH3 | 0.2173913 | 0 | 1 | 0.4057971 | |  |  |  |  | ***Total*** | 0.510467 | |
